# Cancer diagnosis through a tandem of classifiers for digitized histopathological slides

**DOI:** 10.1371/journal.pone.0209274

**Published:** 2019-01-16

**Authors:** Daniel Lichtblau, Catalin Stoean

**Affiliations:** 1 Wolfram Research, Champaign, Illinois, United States of America; 2 Faculty of Sciences, University of Craiova, Craiova, Romania; University of Toronto, CANADA

## Abstract

The current research study is concerned with the automated differentiation between histopathological slides from colon tissues with respect to four classes (healthy tissue and cancerous of grades 1, 2 or 3) through an optimized ensemble of predictors. Six distinct classifiers with prediction accuracies ranging from 87% to 95% are considered for the task. The proposed method of combining them takes into account the probabilities of the individual classifiers for each sample to be assigned to any of the four classes, optimizes weights for each technique by differential evolution and attains an accuracy that is significantly better than the individual results. Moreover, a degree of confidence is defined that would allow the pathologists to separate the data into two distinct sets, one that is correctly classified with a high level of confidence and the rest that would need their further attention. The tandem is also validated on other benchmark data sets. The proposed methodology proves to be efficient in improving the classification accuracy of each algorithm taken separately and performs reasonably well on other data sets, even with default weights. In addition, by establishing a degree of confidence the method becomes more viable for use by actual practitioners.

## Introduction

The best possibility to cure cancer lies currently in its detection from early stages [1], [2], [3]. It is therefore advised that individuals with an increased risk of developing cancer based on history take screening tests from an early age and repeat such tests at certain intervals. In some countries, there are recommendations to take such screening tests for all adults after a certain age, depending on the cancer type [4], [5]. Also, there have been important investments worldwide in acquiring advanced microscopy hardware for hospitals. This leads to an increasing amount of histological slides that have to be analyzed. Computational approaches can support the medical professionals through autonomous learning and direct diagnosis establishment especially by providing a second opinion [6], [7] or even determining evidently benign cases in order to allow the human experts to concentrate on the more problematic slides [8], [9], [10].

Even outside the realm of artificial intelligence driven diagnoses, cancer identification based on digital slides is a highly variable and controversial topic, since diagnostic criteria vary across pathologists and particular case types are very challenging and elicit high variability within and across pathologists [11]. For purposes of this work, the labels established by the human experts are considered ground truth.

The primary focus of this work is on automated grading of a collection of images of histopathological slides of colon tissue. These range from healthy through degree three (most serious). The pursued tasks are as follows.

- Reach a combined, augmented approach from the information provided by six independent classifiers previously considered for the problem [12] through the practical and efficient optimization approach of differential evolution.
- Validate the ensemble method on other histological image data sets.
- Identify the samples that are often misclassified by the proposed hybridized algorithm.
- Define a possibility that allows the human expert (e.g. the pathologist) to separate the collection of data to be classified into a set of samples that can be correctly labeled with a high degree of confidence, and a complementary set of more difficult cases that thus require further attention from the physician.

## 1 Materials

**Fig 1:**
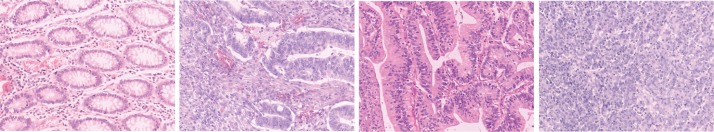
Histological image samples: From left to right, normal tissue followed by cancer of grades G1, G2 and G3.

The image data set comes from the University Hospital of Craiova, Romania, and contains 357 images at 800x600 pixels with 62 healthy (G0) records, 96 of the first grade (G1), 99 of the second grade (G2) and 100 of the third grade (G3). The grades for the samples were established by two pathologists that reached a consensus diagnosis. This diminishes, but does not remove, the possibility that there may be classification errors in some cases where the pathologists must distinguish difficult diagnostic categories. Examples of samples from each class can be observed in Fig 1. Based on the name of the project that put forward the data, it will be further referred in the article as the IMEDIATREAT data set. It was initially introduced in [13] and is available for download [14].

For showing the generalization ability of the proposed approach, two other case studies are considered for the designed combination of classifiers.

One such data set that also refers to colon cancer was put forward through a challenge contest called GlaS that was held at the MICCAI 2015 conference [15], [16]. The contest goal regarded the accuracy of the gland segmentation, not of the actual automated diagnosis. In this respect, along with the raw histopathological images, associated ones with the manual segmented glands are provided. In addition, one of the two diagnosis labels (benign and malignant) is associated with each slide. These can be used when automated diagnosis is desired. The data collection (that will be further on called the GlaS data set, from the contest name) comprises 165 images with a 20x magnification level.

Recently, a large data set of breast cancer histopathology images acquired from 82 patients was introduced in [9] under the name BreaKHis. There are several considered magnification levels, e.g. 40x, 100x, 200x and 400x. For each level in turn, the samples are separated in two classes of approximately 600 benign and 1300 malignant. In total, there are 2480 images of healthy tissue and 5429 of malignant tissue. The methods of extracting the features applied on the BreaKHis are briefly described in the next section and their results will be discussed in the experiments part. To the best of our knowledge, there is no information regarding the number of pathologists that decided the grade for each slide in turn for these two data sets. Both benchmark data sets are available for download; see their respective references for details.

## 2 State of the art in histological image classification

Although the traditional sequence of preprocessing—segmentation—feature extraction—feature selection—classification is still preferred by many studies in image analysis, fully automated classification of cancer histological images has currently emerged as an alternative human-independent methodology. This implies further intervention is not required for pre-annotation of the regions of interest from the pathologists and therefore exempts the human experts from the additional effort of assisting the machine. Recently, more uncommon means of diagnosis have been proposed, like studying the movement of the eyes of the pathologists [17], but that still needs the pathologist to do the classification task. For a very recent and broad literature review about clinical information extraction, including that from histological slides, see [18].

Based only on training pre-diagnosed samples, direct image-level classification for the confirmation or infirmation of the presence of cancer, with additional feature design [19], has been extensively explored.

The authors of [20] use a bag of features approach to gain image representations of basal-cell skin carcinoma slides which are next classified by support vector machines as positive or negative. The study of [21] targets prostate cancer diagnosis through a Bayesian multiresolution system to recognize cancerous regions within digital histopathology slides, with features being selected through an AdaBoost component.

Some very recent attempts are highlighted in the editorial paper of [22]. A competition in mitosis detection from breast cancer histopathology images has compared eleven algorithms submitted for the task [23]. Among them, only two do not use the traditional 4-step sequence, i.e. a multi column max-pooling convolutional neural network [24] and a cascade learning framework with a boosting method [25], both targeting supervised pixel classification (giving the probability of belonging to the mitosis class).

Another convolutional neural network for the classification of breast lesions from histopathological images is used in [26] to perform feature extraction in terms of pattern and distribution, after nuclei had been previously located.

Again texture features, extracted by co-occurence statistics and local binary patterns, are used by a k-nearest neighbor classifier to differentiate between stroma-rich and stroma-poor slides in neuroblastoma patients [27].

First and second order image statistical parameters are given to several classification approaches to distinguish between grades of anal intraepithelial neoplasia from histological slices in [28].

More specifically, concerning the particular case of colorectal cancer slide interpretation, the study of [29] is concerned with images of the 4 cancer classes with 20000 annotated nuclei to be detected. This is done by deep convolutional neural networks with subsequent, separate classification by a neighboring assemble predictor. On the other hand, [30] computes texture feature vectors (on images obtained from 30 colorectal surgically removed tissues) through local histograms and co-occurence matrices and uses them for cancer labeling normal vs. adenocarcinoma by the quasi-supervised statistical learning method.

Other feature identification approaches that are also applied for the BreaKHis data set (which will be further discussed within section 4) are next briefly presented. In [31], the local binary patterns (LBI) operator considers each pixel intensity to compute the distribution of binary patterns by making use of a radius and a number of neighbors. The occurrence histogram of the reached LBI proves to be a good texture descriptor. A recent variant of the method is presented in [32] as the completed local binary patterns (CLBI) and considers the central pixel, sign and magnitude: these bring significant improvement for rotation invariant texture classification. A descriptor, also for texture classification, that is based on quantized phase information of the discrete Fourier transform computed locally in a window for every image position is proposed in [33] and is called local phase quantization (LPQ): the histogram of the resulting image is used as the vector of features. The gray-level co-occurrence matrix (GLCM) represents a classical method [34] that is still widely used in the present and assumes the calculation of the *Haralick* parameters. A morphological measure for cell phenotype image classification is introduced through the threshold adjacency statistics (TAS) in [35]. Thresholding is applied to the image and, on the subsequent binary image, for each white pixel, the number of adjacent pixels that are also white are considered to reach some statistics that proved to be important in the classification process. A parameter-free version of TAS (PFTAS) is introduced in [36]. The class structure-based deep convolutional neural network (CSDCNN) [37] is a recent successful non-linear representation learning model that also abandons feature extraction steps. Instead it automatically learns semantic and discriminative hierarchical features from low-level to high-level. Another very recent deep classifier is represented by the supervised intra-embedding method with a multilayer neural network model, followed by a CNN [38].

Except for one classifier of the proposed ensemble, these use the numerical features extracted by a CNN for classification. For none of them are separate landmark or feature considerations provided. As the results will reveal in the following sections, the fact that one classifier has a different manner of dealing with the slides proves to be important, since it will exert a large influence over the ensemble decision. Once several well-performing classification models are constructed, the resulting probability estimations for the four cancer grades on the validation samples can be joined in an optimized way that allows complementarities to achieve a boosted level of accuracy. A first naive attempt to reach a better performing ensemble has been recently designed in [12], by simply doubling the weight of a new Fourier trig transform with principal component analysis classifier which seemed to behave differently from the the other machine learners within the classification process.

## 3 Methods

A combination of 6 machine learning techniques (5 state-of-the-art methods and a relatively new approach) is employed for the histopathological image classification task. The images are transformed using the AlexNet CNN [39] into numerical vectors, using the pre-trained weights and without fine tuning. Each vector has a size of 1024 numerical features extracted from the CNN.

The outputs of these involved approaches, in the form of test probability estimations for each outcome, are next combined to provide a further enhancement in accuracy. An optimization process by differential evolution takes into account the degree to which a classifier is wrong and penalizes the mistakes proportionally.

The 5 classifiers were selected from types of machine learners exhibiting different properties, i.e. random forests (RF) [40], nearest neighbours (kNN) [41], logistic regression (LR) [42], naive Bayes (NB) [43] and support vector machines (SVM) [44]. A novel Fourier trig transform with principal component analysis (FTT+PCA) approach [45] was additionally included in the pool of methods, due to its competitive results. Each classifier is independently trained and applied to the test set.

In a previous attempt [12], the weight of the last technique was doubled within the voting process for a common prediction of the chosen classifiers which led to improved accuracy. The aim of the current work is to further and automatically enhance the classification output, as well as provide some degree of confidence with respect to the classified samples. Information regarding how large the errors are, meaning how far the classified samples from the ground truth, will also be found. Moreover, those samples are identified that are most frequently mistaken (which gives some insight into what might cause erroneous classifications).

The outputs for each of the 6 individual classifiers are considered in the form of probabilities, so instead of deriving the direct label for a test sample, the result displays four probabilities, one for each class of the problem. In this way, for the cases that are harder to discern, the methods do not provide the direct class, but more information regarding the way the decision is split between the classes is given. The most direct manner to reach the decision for a test sample would be to allow for an equal importance to each method and consider the class with the maximum sum or average for each possible grade over the probabilities reached by every single classifier. The grade that has the highest value would then be the determined label for the sample to be classified. However, as some classifiers may have their results highly correlated, they would dominate the labeling for most of the test samples, and this would result in an accuracy that is not significantly higher than the algorithm that performs best. To counteract this effect, weights are used to influence how much each classifier counts for the final decision, in the spirit of other weighted combinations of classifiers [46], [47].

The task of determining these weights is performed by an optimization metaheuristic, i.e. a Differential Evolution (DE) algorithm [48], [49]. DE proved to be a top performer in most of the competition series organized by IEEE Congress on Evolutionary Computation (CEC), globally surpassing all the other search paradigms for single objective, constrained, dynamic, large-scale, multi-objective, and multi-modal optimization problems [50]. Additionally, personal experience [51], [52] has shown that DE is robust, readily handles integer-valued objective functions, and has few control parameters which makes it easy to use. As will be seen, the obtained results justify this choice.

The classification process, with particular reference to the optimization for the weights, can be summarized as follows. In the first step, the classification models are constructed on the training examples. Once the 6 models are built, their prediction results are computed for the validation samples. The labeling for a sample is in the form of four probabilities, corresponding to the four possible categories, and it is provided for each of the 6 methods. Optimal weights to balance the outputs of the 6 classifiers are determined by DE on subsets of the validation set. The best weighting is eventually applied to the model probabilities of the complementary subsets to assess the final prediction accuracy.

The candidate solutions used by the DE have 6 variables that represent the weights for the same number of considered classifiers. The interest lies in relative weighting, so they are not constrained to sum to unity. Nevertheless, in order to still have control over the variables, boundaries are set for each of them in the interval [0, 1]. Another imposed constraint is that the overall sum of the weights should be in [0.5, 2]. Thus, the sum of weights has precise boundaries, and situations in which all weights are very small (close to zero) or very high (close to 1) are avoided. The fitness function penalizes errors that are worse than off-by-one class in order to diminish the amount of samples that are mistaken by a larger extent. It is given in Eq (1): **w** is the vector of weights, *n* is the number of samples in the validation set, *dc*_*i*_ and *ac*_*i*_ are the determined and the actual classes for sample *i*. The determined class *dc* for a sample is considered to be the label for which the highest value is obtained when applying the weights for the probabilities of the 6 classifiers.

$$\begin{array}{c} f\left(w\right)=\sum_{i=1}^{n}|dc_{i}\left(w\right)-ac_{i}{|}^{2} \end{array}$$ (1)

## 4 Experimental results

Two experiments are undertaken.

The first one is represented by a usual classification task. Models for each individual classifier are constructed based on the training samples and are used to classify the validation and test sets. Next, a metaheuristic is used for searching the values that weigh the probabilities of the six classifiers with the aim of boosting the classification accuracy for the validation set. A final overall accuracy is calculated on the remaining test set.

The second experiment uses the results from the first one to establish the trade-off between the fraction of “trusted” results vs. failure rate of trusted results (ideally, one wants the fraction of trusted results to be high, with few or no failures falling into that category).

**Fig 2:**
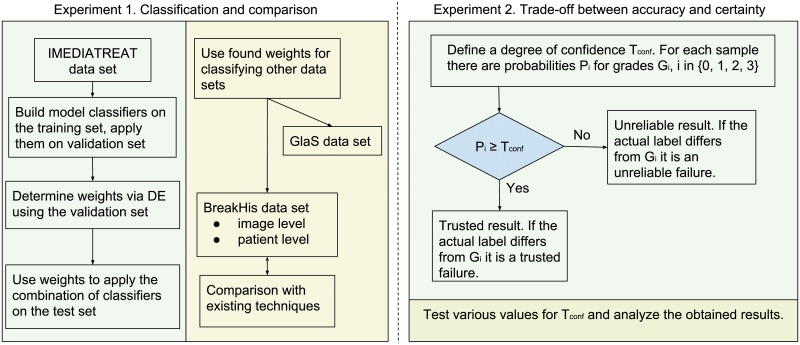
Organization of the experiments.

Fig 2 shows the overall flow of the experiments.

The implementation of all algorithms is made in Wolfram Mathematica, whose version 11.3 integrates automated functions for the employment of popular machine learning approaches for classification using CNN-extracted features in computer vision [53]. The Mathematica code is available at https://github.com/catalinstoean/DE-hybridization-classifiers.

### Experiment 1: Ensemble of the various classifiers

The samples set aside from the training are now split into validation and test subsets. The first is used to search for an optimal manner of combining them, i.e. finding classifier weights through a metaheuristic to improve classification accuracy on this learning data. Monte Carlo cross-validation [54] is used to generate the splits. The resulting weights are applied to the complementary sets of examples in order to help assess the test prediction accuracy.

Besides being tested on the IMEDIATREAT data set, the weights revealed by the proposed ensemble classifier are also applied to the GlaS and BreaKHis collections. On the latter, the obtained results are compared to the ones obtained by other state-of-the-art classifiers [9].

#### Pre-experimental planning

In the initial tests, direct classifications (rather than probability scores) for each sample were considered for the optimization process. Although the results were encouraging and better than the outputs of any individual classifier, it was later decided to include more information in the process. Specifically, this meant taking into account the four-class probabilities for each sample.

Within the same pre-experimental stage it was decided to penalize more heavily those misclassifications that went outside their correct class by more than one disease grade, as shown in Eq (1): by squaring the distance between the found class and the actual one, the penalization is increased when the error is higher. This type of evaluation gave cleaner results, both with respect to the degree of misclassification and also to the actual classification accuracy results, as opposed to those obtained when all incorrect assessments counted equally.

Several options were tried for setting the DE to boost the classification accuracy. Initially it was thought that the low probabilities might complicate the overall decision and a cutoff threshold was taken into account. Thus, all the probabilities below that threshold were clipped to 0. Such a cutoff threshold was also considered in the candidate solution for the DE in order to discover the most appropriate value. A variant that considered specific cutoff thresholds for each classifier was also tried during pre-experimentation. Although at first glance, the classification results obtained appeared to be slightly better, the differences were not statistically significant and it was decided to keep the simpler approach that evolves only the weights for the classifiers.

#### Task

The task is to find a way to combine the class probability outputs of the classification models on the validation samples, which provides an accuracy significantly better than that of the standalone classifiers. Further, it is desired to understand why the computed optimal weights work better than others. Finally, the results of the proposed ensemble classifier are compared to those of other powerful learners on the BreaKHis data set.

#### Setup

Monte Carlo cross-validation [55] is employed: 40 random splits are conducted, each time holding 67% of the samples for training, with the rest for validation and test. The next phase uses random subsets comprised of half these 33% trials in order to compute optimal weights for combining the scores from the separate methods. The most promising ones are then checked on the complementary subsets (i.e. test sets). The one that extrapolates best (as gauged by lowest error count on the complementary set) is chosen as the overall optimal weighting. The outcomes for the validation and test samples give both the individual classifications and the probabilities, i.e. for each sample, there are four probabilities that refer the level of belonging to each of the four classes of the problem.

The 5 well established classifiers are implemented in Mathematica using the *Classify* function. They extract characteristics from slides based on the AlexNet determined features, without guidance and are used with their default settings. No further parameter tuning was performed, since the results were already acceptable. A splitting criterion given by the minimal entropy and 200 trees are considered for RF. The Euclidean distance and 2 neighbors are used for kNN. For LR the regularization coefficients are L1 = 0 and L2 = 10. There are 1024 features extracted for NB.

The DE-based optimization is performed 100 times, each time optimizing validation results for a random subset of 20 of the 40 original trials. The quality is assessed both by error rates in these validation subsets, and, more importantly, by how well the same weights performed on the 20 trials not used in the given optimization run. In each run the population size of the DE is set at 50, and the DE will stop at 1000 iterations if convergence does not happen sooner. The crossover probability is set to 0.5 and the differential weight to 0.6. Various other values were considered and, while several delivered good results, the parameters indicated above reached the best found solution in a reasonable amount of time.

The fitness function penalizes the errors that are worse than off-by-one. The amount by which the classes are mistaken is squared. The fitness function has to be minimized, since the classification error needs to be as low as possible.

All the settings described above are tested against the IMEDIATREAT data set as this is the problem of primary interest in the current research. It is of course also desirable to show that this methodology, and even the computed optimal weights, have applicability to other data sets of comparable composition. For this purpose two other data sets are included in the current experiment. It must be noted that in [9] the recognition rates are computed at the patient level instead of the image label. Accordingly this type of computation is adopted for the BreaKHis data here as well, beside the usual image level count, in order to have comparable results. In short, this implies computing for each patient *P* the ratio between the number of correctly identified images that correspond to *P* and the total number of images of *P*. Then, the final recognition rate will be computed as the sum of all the patient scores divided by the number of patients. The DE is applied to search the optimal weighting for each data set in turn to be able to compare the various sets of weights. Additionally, the weights for IMEDIATREAT are applied for the BreaKHis data set to observe how well they perform from one problem to another.

#### Results

Table 1 shows the overall classification accuracy for IMEDIATREAT data set together with the standard deviation, minimum, maximum, and confusion matrix of the combined approach as obtained from 40 repeated runs. Table 2 illustrates several statistical measurements per class for the same runs. Table 3 shows the individual accuracy results for each classifier and for each task in turn.

**Table 1:** Results on the IMEDIATREAT data set. Classification accuracy and confusion matrix in percents (true on the columns and predicted on the rows) for the combined approach.

Accuracy	St. dev.	Min	Max	Predicted	True			
					G0	G1	G2	G3
98.29	1.24	93.28	100	G0	17.39	0.00	0.02	0.00
				G1	0.02	26.85	0.88	0.00
				G2	0.23	0.02	26.39	0.06
				G3	0.00	0.02	0.44	27.67

**Table 2:** Statistical measurements per class in percents for the IMEDIATREAT data set for the combined approach.

Class	Specificity	Precision	Recall	F1 score	Balanced accuracy
G0	99.7	98.6	99.9	99.2	99.8
G1	99.9	99.8	96.7	98.3	98.3
G2	98.2	95.2	98.8	97	98.5
G3	99.9	99.8	98.4	99.1	99.1

**Table 3:** Classification test accuracies in percents for the individual classifiers and for each data set in turn.

Data set	RF	kNN	SVM	LR	NB	FTT+PCA
IMEDIATREAT	90.5	93.89	95.04	94.2	86.76	90.46
GlaS test set A	93.33	83.33	90	90	91.67	58.33
GlaS test set B	100	95	100	90	100	65
BreakHis 40x	78.9	76.55	81.61	79.44	72.23	76.78
BreakHis 100x	81.09	77.4	84.47	82.89	79.14	77.9
BreakHis 200x	84.15	82.46	86.67	84.77	83.24	83.19
BreakHis 400x	82.28	79.53	83.15	82.64	81.52	81.52

**Fig 3:**
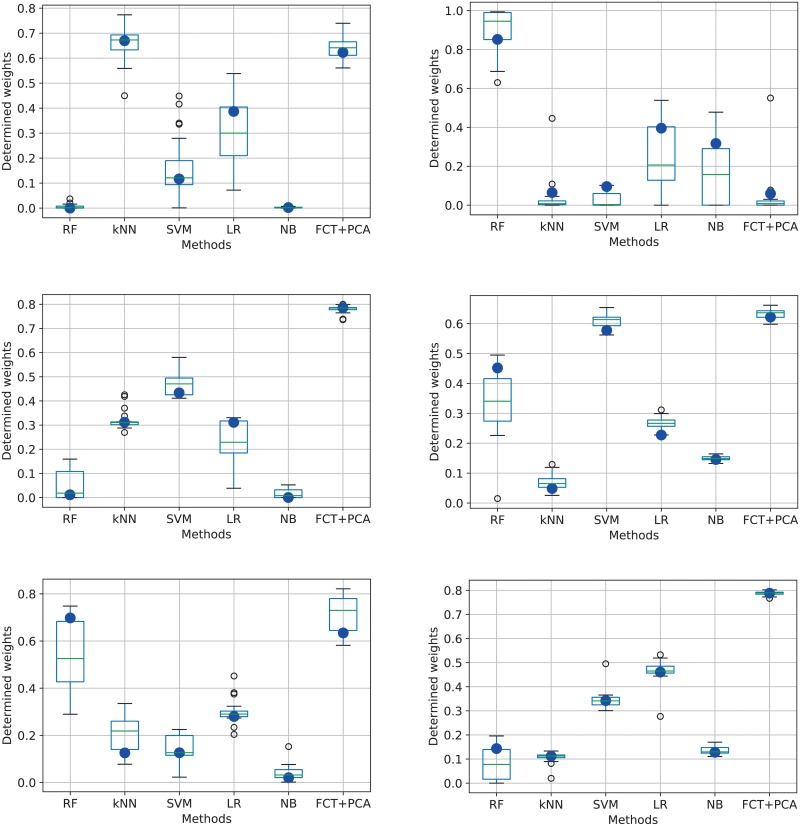
Box plots showing the variation in weight for each classifier in turn as determined by the final population of the DE. First row corresponds to the weights found for IMEDIATREAT and GlaS (test set B) data sets, second row for 40x, 100x for BreaKHis, while the last row show the values for 200x and 400x respectively. The blue filled circle represents the best configuration result.

**Fig 4:**
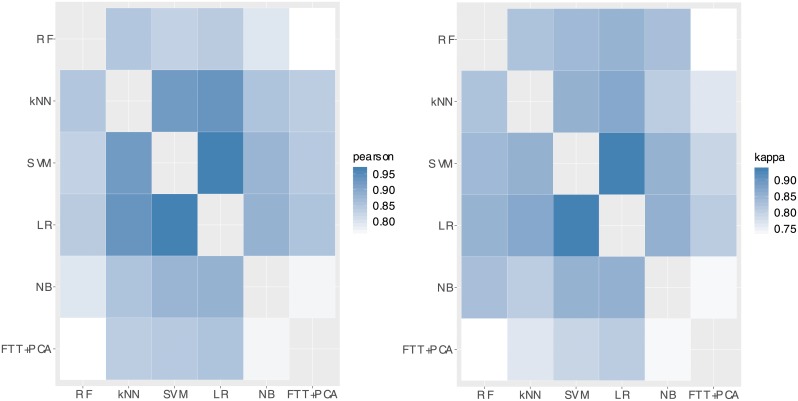
Correlations between outputs of the individual classifiers. On the left, Pearson product-moment correlation coefficients takes into account probabilities outputs, while on the right Cohen’s kappa coefficients are based on the actual test accuracy results. Darker colors correspond to higher correlation.

Fig 3 outlines the box plots with the weights as discovered by the DE for each method in turn. The best found configuration is indicated with a blue filled circle. Fig 4 illustrates how correlated the results of the individual classifiers for the IMEDIATREAT data set are, taken two by two.

Table 4 illustrates the classification results obtained for the GlaS data set using the weights discovered by DE for IMEDIATREAT and BreaKHis data sets.

**Table 4:** Comparison on the benchmark data sets. Applications to GlaS and BreaKHis data sets and comparison to results obtained by other techniques.

GlaS data set				
Method	Image level recognition (%)			
	Test Set A		Test Set B	
Proposed DE ensemble	96.66		100	
BreaKHis data set				
Method	Image level recognition (%)			
	40x	100x	200x	400x
Proposed DE ensemble	83.9	86	89.1	86.6
CNN strategy 4	89.6	85	82.8	80.2
CNN Sum combination	85.4	83.3	83.1	80.8
Fisher CNN	87.7	87.6	86.5	83.9
CSDCNN	95.8	96.9	96.7	94.9
Method	Patient level recognition (%)			
	40x	100x	200x	400x
Proposed DE ensemble	85.6	87.4	89.8	87
CLBP+SVM	77.4	76.4	70.2	72.8
GLCM+SVM	74.0	78.6	81.9	81.1
LBP+SVM	74.2	73.2	71.3	73.1
LPQ+SVM	73.7	72.8	73.0	73.7
PFTAS+SVM	81.6	79.9	85.1	82.3
CNN strategy 4	88.6	84.5	83.3	81.7
CNN Max combination	90	88.4	84.6	86.1
Fisher CNN	90.2	91.2	87.8	87.4
CSDCNN	97.1	95.7	96.5	95.7

**Fig 5:**
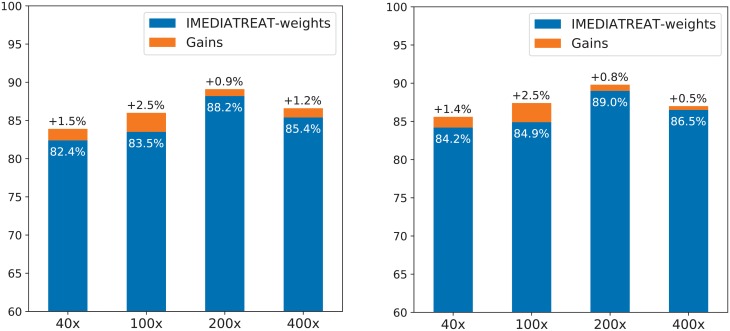
Test classification accuracy for BreaKHis when using weights discovered by DE as applied to the IMEDIATREAT data set, and the gains in percent when the DE optimizes the weights directly on BreaKHis. Left plot contains image level recognition, while the right one illustrates patient level recognition.

Fig 5 illustrates the classification results for BreaKHis when using the weights discovered for IMEDIATREAT and what are the gains when the DE is applied for that particular data set.

### Experiment 2: Establishing a degree of confidence to separate data into trusted and non-trusted

Despite the accurate results of the proposed combined approach, the confusion matrix shows that there are some cases that are mistaken even by two grades. In this respect, a physician would be interested in a subset of data that would be known with a high level (preferably 100%) of certainty as correctly classified. This way, the physician could ideally count on the classifier to correctly assess that subset of data and she or he would concentrate on the other samples that are harder to classify. Or, in a more realistic scenario, the pathologists could receive the classifier’s output subsequent to their interpretation, such that an automated judgment would be complementary, a welcome second opinion [56]. The current experiment is dedicated to deciding such subsets of trusted or non-trusted samples. To some extent, this separation of samples resembles concepts like sensitivity and specificity, but the current study does not deal with a binary classification problem and the interest lies in finding a subset of the data set where the error is acceptable. Additionally, herein the term “trusted” refers only to the assignation of those samples to a class with a high probability, and not to those that are also correctly classified. Nevertheless, a subsequent analysis measures the amount of samples that are wrongly classified within the trusted set. Going further with the second opinion scenario, associating a degree of confidence for each slide facilitates the prioritization and the review for the human expert.

#### Pre-experimental planning

The aim was to make use of the class probabilities for each sample and decide that an image is assigned to a grade only when the values go beyond a certain threshold. The initial attempt was to establish a value for the degree of confidence (e.g., 0.8) and separate the data into trusted and non-trusted results. Such a threshold would imply that only the samples that are assigned to a class with a probability larger than 0.8 will be established as *trusted*. However, the output very much depends on the threshold and it was instead decided to vary its values and obtain a Pareto front with a tradeoff between percent trusted (desired to be high) and trusted failures. The latter correspond to false positives and false negatives in the terminology of the binary case scenario, and thus should to be as low as possible.

#### Task

Establish sets of trusted and non-trusted samples with respect to a degree of confidence and evaluate the amount of the trusted data that is still wrongly labeled.

#### Setup

This experiment uses the weighted scores as determined from the prior experiment. It is assumed that each sample has a largest probability *p*_*i*_ assigned to a grade G*i*, where *i* ∈ {0, 1, 2, 3}. The threshold for the degree of confidence, denoted further *T*_*conf*_, will be varied from 0.5 to 1 in steps of 0.05. Depending on the relation between *p*_*i*_ and G*i* and whether the samples are correctly classified or not, they will be depicted as:

- *Trusted results*: samples for which *p*_*i*_ ≥ *T*_*conf*_.
- *Trusted failures*: samples for which *p*_*i*_ ≥ *T*_*conf*_ and the actual label is different from G*i*. This set should ideally be null. If the size of this set is subtracted from the number of *Trusted results*, the number of samples that are both trusted and correct is obtained.
- *Unreliable results*: samples for which *p*_*i*_ ≤ *T*_*conf*_. These results together with the trusted results represent the entire set of samples.
- *Unreliable failures*: samples for which *p*_*i*_ ≤ *T*_*conf*_ and the actual label is different from G*i*. When the size of this set is subtracted from the number of *Unreliable results*, the number of samples that are correctly classified, but not trusted, is achieved.

#### Results

**Fig 6:**
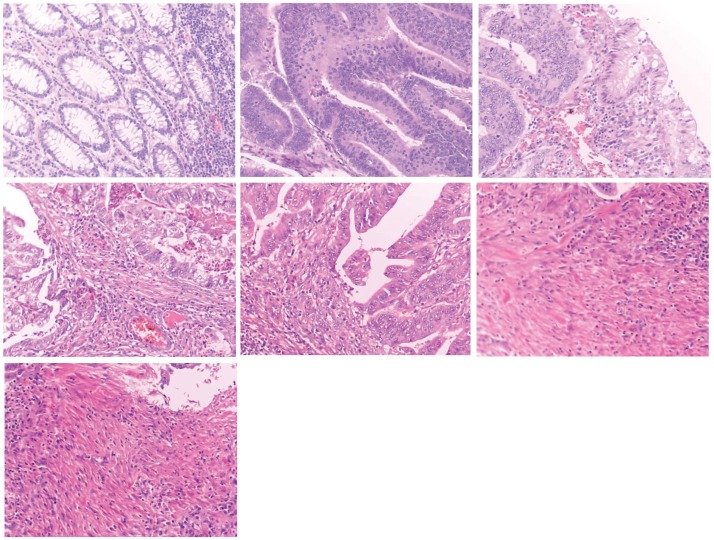
Sample images that are wrongly classified by the proposed classifier. Their assigned grades are G0 (image from the first row and first column), G1 (first row, second column) and G2 for the rest (since G2 images are those that are most commonly misclassified).

Fig 6 shows the images that under repeated runs are wrongly labeled by the proposed combined approach.

**Fig 7:**
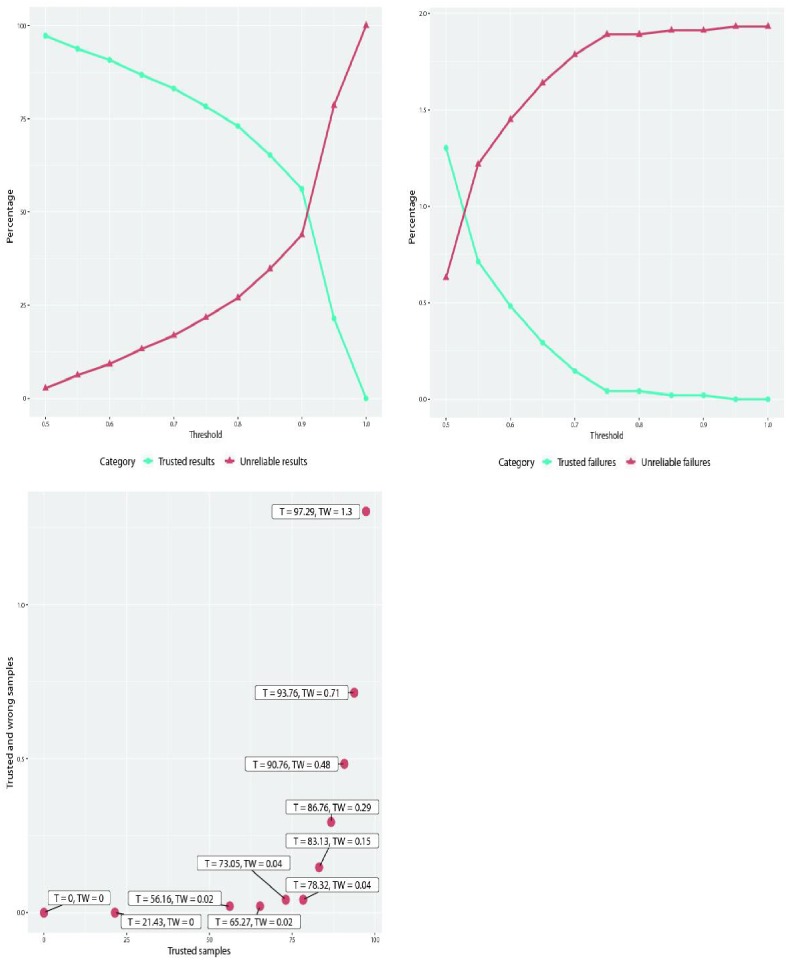
The confidence threshold is varied from 0.5 to 1. The trusted failures from the first row right plot are included in the trusted results from the first row left plot. Analogously, unreliable failures are part of the unreliable results. The plot from the second row illustrates the Pareto front between the trusted samples (denoted by “T” in the labels) and the trusted but wrongly classified (denoted by “TW”): both axes are in terms of percents.

Fig 7 shows how the sizes of the sets described above are changed when the *T*_*conf*_ threshold is varied.

## Discussion

### Ensemble of the various classifiers

There are two different approaches that were previously applied for this data set: one represents a multiple step approach that performs pre-processing, image segmentation, extraction of numerical features, selection of a subset of most discriminative characteristics and eventually classification on the resulting attributes. Such an approach achieved a maximum classification accuracy of 83.94% [57]. A second approach refers to a CNN containing 5 convolutional layers that had a performance of 91.44% [58], and was further tuned in [59] reaching 92% in classification accuracy.

The classification results of the combined approach on the IMEDIATREAT data set, as shown in Table 1, are significantly better than those of the previous naive design of doubling the weight of the Fourier transform approach, which led to 95.65% correct labeling [12].

Besides the confusion matrix in Table 1, Table 2 brings more insight concerning the separation between the different classes for IMEDIATREAT data set: the slides that correspond to the healthy tissues on the one hand and the ones that correspond to G3 on the other hand are better delineated from the rest with respect to all the considered metrics, while the slides that are more difficult to distinguish come from G1 and G2. This observation is especially sustained by the values of the specificity, showing that there are only few images that do not have this condition and are labeled as such.

In order to achieve each plot from Fig 3, the DE is run 100 times, each run using a random subset of 20 of the 40 trials to optimize the weights, with the weights then tested on the remaining 20. The box plots are computed from the weights contained in the best candidate solutions. A general observation is that FTT+PCA has a relatively high weight over all data sets, except for GlaS, where it performs poorly. In general, the boxes are relatively small, meaning that the degree of dispersion is relatively low, so the weights are close to each other. The exception is represented in general by RF (which is kept to zero only for IMEDIATREAT), where a large variety of weights leads to good results. Besides FTT+PCA, SVM and LR are the models that have high influence for most of the problems.

However, it is important to notice that the weights plotted in Fig 3 are not always in direct agreement with the individual accuracies of the chosen classifiers in Table 3. In order to understand this behavior, the Pearson product-moment correlation coefficients were computed for each pair of methods by taking into account the class probabilities outputs on the test sets for IMEDIATREAT data set and the Cohen’s kappa coefficients based on the actual test accuracies. The results are illustrated in Fig 4. By analyzing it in conjunction with the first plot in Fig 3, they show that (1) those methods that misclassified different test samples as opposed to the other techniques got higher weights relative to their individual performance and (2) methods gave similar labeling to others (not only as concerns the actual labels, but also the probabilities for each grade in turn) got lower weights.

In summary, the generated weights are not always proportional to the accuracies obtained by each of the six methods, as the DE finds an optimum that takes into account the level of disagreement of each classifier with the other classifiers. For IMEDIATREAT, DE therefore puts again a very high weight on the FTT+PCA, as it was also empirically observed to be efficient in the earlier naive approach [12]. The same high weight can be found for it for BreaKHis in all four magnification levels. The optimized approach results on the IMEDIATREAT data substantially surpassed the initial expectations as concerns the achieved classification accuracy, boosting the correctness rate by 3 percentage points.

Table 4 contains the results of the proposed method for the other two public data sets, i.e. GlaS and BreaKHis using the weights discovered by DE in the best configuration, the one illustrated with a blue circle in Fig 3. As seen in Table 4, the recent deep learning classifier CSDCNN reaches the most accurate results as compared to the other state-of-the-art methodologies. However, a distinct protocol is considered for CSDCNN, i.e. different splits and especially a larger training set. Excepting this method, the proposed methodology reaches the best results for 200x and 400x in the image level results and for 200x for the patient level computations, while in other cases it performs close to the best.

Although for the IMEDIATREAT and BreaKHis data sets FTT+PCA has the highest influence in the ensemble (for the former it is very close at the top to the one of kNN), it fails to deliver good results for GlaS on its own and accordingly it was taken out by DE from the ensemble. The poor results are probably reached due to the very small size of the GlaS data set, as opposed to the other two.

The DE ensemble approach was employed for finding weights specific to the individual classifiers that work best for each different image data set. We were interested however in testing how the weights discovered for IMEDIATREAT data set would perform if transferred to the BreaKHis data set. Fig 5 illustrates the obtained results and the actual gains when the DE is used specifically for that data set. As it can be noticed, the gains are significant in general, but the IMEDIATREAT weights already lead to a decent result, better than most of those delivered by many other methods illustrated in Table 4.

As concerns the running times, for the 40x magnification BreaKHis data set, the training of all classifiers on 1338 images takes in total 17.7 minutes. Also as part of the training, DE is further applied to find the weights and this lasts 125 seconds. For testing the entire ensemble on one image, the time is 0.69 seconds. The tests are performed on a PC running Ubuntu Linux, 2.9 Ghz CPU and 16 GB RAM.

### Degree of confidence

As quantified in Fig 7, the higher the threshold for *T*_*conf*_ is, the smaller the set of samples that are trusted. Similarly, the amount of trusted failures, i.e. the samples that are put in the trusted set but the classifier is wrong about them, is decreased, as desired.

Out of 92 missed guesses of the hybridized method, 55 are generated by the images from Fig 6. The anarchic structure from G3 has a high similarity degree especially with the last two slides. Another fact that should be considered is that the data set is obtained by cropping the slides from larger images that contain borderlines between healthy tissues and unhealthy ones of grades 1, 2 or 3, following the acknowledged procedure in [60].

By analyzing the blue lines in the two plots from Fig 7, one can notice that in the situation when *T*_*conf*_ is equal to 0.95, the trusted failures is 0, and there are still 21.43% results that are labeled correctly. When *T*_*conf*_ is 0.85, 1 sample out of 4760 is incorrectly labeled and 65.27% (3107 samples out of 4760) are both labeled correctly and trusted to be so with respect to *T*_*conf*_. The methodology would thus surely help human experts to establish the grading for histopathological images. By setting the *T*_*conf*_ to around 0.85, the pathologists would in principle have more than half of the samples classified and they could focus on the cases that are more difficult to distinguish.

## 5 Conclusions

The current research is focused on improving the classification accuracy on the IMEDIATREAT collection. It is comprised of 357 histopathological slides that are separated into four different classes: healthy or cancer of grades 1, 2 or 3. There are six classifiers that are tested on the data set: they learn from a training set consisting of 2/3 of the entire data collection and are tested on the remaining 1/3 samples. The classification accuracies of these six approaches are superior or similar to the previous attempted techniques on the same data set. However, the research goes further and combines the six classifiers, establishing weights for their significance in order to further boost the accuracy. The values for the weights for the classifiers are discovered (that is, optimized) via a differential evolution approach, using a fitness function that further penalizes the errors where the classes are wrong by more than one grade.

The ensemble approach is successfully applied to two other data sets that consist also of histopathological images, a small one, GlaS, and a very large one, BreaKHis. The latter could be seen as consisting of 4 large data sets of different magnification levels. The ensemble with the weights as discovered by the DE for the IMEDIATREAT data reaches very competitive results on the BreaKHis data set, but still, using the DE optimizer for that specific data set boosts the accuracy results by approximately 1.5% on average.

It is interesting to observe that it is not the classifiers that perform best that are selected as the most important ones by the DE optimizer. As observed in the first experiment, a classifier that reaches a classification accuracy usually below average counts the most in general and it is also observed that it does not agree at a high extent with the other classifiers as reflected by a Cohen’s kappa test.

A degree of confidence is further defined to separate the classified samples into trusted (with respect to their classification) or unreliable. The classification problem that is dealt with is a very delicate one, so it is perhaps more important to divide the histopathological slides to be tested into a set that the classifier can distinguish very well and a set where there are doubts. The latter subset could be regarded as in need of more human expertise. Without a doubt, the pathologists will never be replaced, but a high amount of work could be set by establishing the cases that are very clear, or at least be afforded a strong second opinion. This could provide pathologists with a means for prioritizing samples that would need further scrutiny.

The proposed approach combining the six classifiers proves to be very strong with respect to the classification accuracy, especially as compared to the individual results of the six methods. By taking into account the probabilities for each grade in turn, the DE manages to balance the strengths of each classifier with correlations between methods. In setting a larger weight for the FTT+PCA method, the weighted ensemble often reaches the correct labeling where FTT+PCA is mistaken, by counting on the combined weight of those classifiers that are correct, and takes the correct FTT+PCA label where some other classifiers may have their best choices spread among several possibilities. This finding may have future impact on other classification problems as well.
